# *De novo* transcriptome assembly and novel microsatellite marker information in *Capsicum annuum* varieties Saengryeg 211 and Saengryeg 213

**DOI:** 10.1186/1999-3110-54-58

**Published:** 2013-11-21

**Authors:** Yul-Kyun Ahn, Swati Tripathi, Young-Il Cho, Jeong-Ho Kim, Hye-Eun Lee, Do-Sun Kim, Jong-Gyu Woo, Myeong-Cheoul Cho

**Affiliations:** Vegetable Research Division, National Institute of Horticultural & Herbal Science, Rural Development Administration, Suwon, 440-706 Republic of Korea

**Keywords:** *Capsicum annuum*, Next generation sequencing, Transcriptome profiling, Molecular markers, Simple sequence repeats

## Abstract

**Background:**

Pepper, *Capsicum annuum* L., Solanaceae, is a major staple economically important vegetable crop worldwide. Limited functional genomics resources and whole genome association studies could be substantially improved through the application of molecular approach for the characterization of gene content and identification of molecular markers. The massive parallel pyrosequencing of two pepper varieties, the highly pungent, Saengryeg 211, and the non-pungent, Saengryeg 213, including *de novo* transcriptome assembly, functional annotation, and *in silico* discovery of potential molecular markers is described. We performed 454 GS-FLX Titanium sequencing of polyA-selected and normalized cDNA libraries generated from a single pool of transcripts obtained from mature fruits of two pepper varieties.

**Results:**

A single 454 pyrosequencing run generated 361,671 and 274,269 reads totaling 164.49 and 124.60 Mb of sequence data (average read length of 454 nucleotides), which assembled into 23,821 and 17,813 isotigs and 18,147 and 15,129 singletons for both varieties, respectively. These reads were organized into 20,352 and 15,781 'isogroups’ for both varieties. Assembled sequences were functionally annotated based on homology to genes in multiple public databases and assigned with Gene Ontology (GO) terms. Sequence variants analyses identified a total of 3,766 and 2,431 potential (Simple Sequence Repeat) SSR motifs for microsatellite analysis for both varieties, where trinucleotide was the most common repeat unit (84%), followed by di (9.9%), hexa (4.1%) and pentanucleotide repeats (2.1%). GAA repeat (8.6%) was the most frequent repeat motif, followed by TGG (7.2%), TTC (6.5%), and CAG (6.2%).

**Conclusions:**

High-throughput transcriptome assembly, annotation and large scale of SSR marker discovery has been achieved using next generation sequencing (NGS) of two pepper varieties. These valuable informations for functional genomics resource shall help to further improve the pepper breeding efforts with respect to genetic linkage maps, QTL mapping and marker-assisted trait selection.

**Electronic supplementary material:**

The online version of this article (doi:10.1186/1999-3110-54-58) contains supplementary material, which is available to authorized users.

## Background

Pepper (*Capsicum* spp., Solanaceae), is an important vegetable crop mostly used as spice, condiment, medicine, vegetable and a significant source of vitamin A and C (Bosland and Votava 2000), throughout the world. Originated and initially domesticated in the Americas, the genus includes 38 species with 5 (*C. annuum*, *C. chinense*, *C. frutescens*, *C. baccatum*, and *C. pubescens*) domesticated ones (Hill et al. 2013), having *C. annuum* as the most economically important one (Bosland and Votava 2000; Perry et al. 2007). It has been used as a model organism for classical and molecular genetics analyses very similar to the other Solanaceous member tomato. The detailed genetic, mapping, comparative genomics and gene-based association studies facilitate the identification of quantitative traits loci (QTLs) and subsequent identification of genes via map-based cloning strategies to understand the genetic control of various traits (Xu et al. 2003; Jun et al. 2008). These studies are enabled by the genome-wide molecular characterization of the germplasm (Lee et al. 2004; Yi et al. 2006; Kim et al. 2008) and require enough molecular markers for the production of dense linkage and association maps to identify these QTLs. Numerous genetic markers have been identified and mapped in the recent past (Prince et al. 1995; Ben Chaim et al. 2001; Hernandez-Verdugo et al. 2001; Lefebvre et al. 2001; Rao et al. 2003; Paran et al. 2004; Oyama et al. 2006; Kim et al. 2008) and Simple Sequence Repeats (SSRs) and Single Nucleotide Polymorphisms (SNPs) have been found as the most attractive ones for these studies (Lee et al. 2005). SSRs (Microsatellite markers) are important because of their locus-specific codominant and multiallelic nature, significant abundance in the genome, and high rates of transferability across species (Saha et al. 2006; Aggarwal et al. 2007). These have already been widely used in molecular mapping of important genes in many organisms, marker-assisted selection (MAS) in breeding, genetic diversity and distance evaluation, genome-wide association analysis, and comparative genetics (Mian et al. 2008; Cavagnaro et al. 2010; Csencsics et al. 2010; Dutta et al. 2011; Jun et al. 2011). Despite the numerous available markers, the whole genome association studies in pepper are not possible as the marker density is not enough to target candidate genes underlying a QTL region and conduct association mapping for complex traits (Livingstone et al. 1999; Minamiyama et al. 2006; Barchi et al. 2007; Wu et al. 2009; Truong et al. 2010; Kong et al. 2012). These studies require a large number of markers and cost effective genotyping technology, where lack of markers covering the whole genome stands as the major limitation to the development of high throughput genotyping assays. The rapid identification of these markers associated with complex, economically important traits in crops has been hindered by the lack of whole genomic sequence, high-resolution maps and cost-effective platforms for high density genotyping. This eventually restricts the commercial application of these genomic resources for gene discovery and molecular breeding.

Recent advances in next generation sequencing (NGS) technologies have created unprecedented opportunities for generating genomic information in even previously uncharacterized systems (Logacheva et al. 2011; Lu et al. 2011; McDowell et al. 2011; Sloan et al. 2012). The process of whole genome sequencing, rapid identification and annotations of gene sequences (Mizrachi et al. 2010; Garg et al. 2011), novel and alternatively spliced genes (Roberts and Smith 2002) and the determination of the variations in nucleotide sequences provides new platform for gene expression analysis (Ameline-Torregrosa et al. 2006; Kim et al. 2006; Cohen et al. 2010) and identification of regulatory elements, target critical genes and enormous polymorphic molecular markers motifs (Gore et al. 2009; Tangphatsornruang et al. 2009; You et al. 2011; Zalapa et al. 2012; Zhu et al. 2012). With these detailed informations, QTL fine mapping and map-based cloning of economically important genes which require development of high density molecular markers in QTL regions become more achievable in identification of genes for important and complex traits (Ben Chaim et al. 2001; Mian et al. 2008; Lee et al. 2009). The transcriptome assembly of this economically important pepper crop is a major requirement in order to generate high-quality gene based molecular markers that are an important resource for determination of functional genetic variations and could be used in breeding programs. Therefore, aiming the transcriptome assembly and cost effective identification of SSR markers, first transcriptome profiling of two varieties of pepper, *C. annuum;* the 'highly pungent’ Saengryeg 211 and 'non-pungent’ Saengryeg 213 (Jung et al. 2006) using 454 pyrosequencing technology with emphasis on microsatellite markers discovery has been performed in the present study.

## Methods

### Plant material and cDNA preparation

Mature fruits from two varieties of pepper *C. annuum* L., Saengryeg 211 and Saengryeg 213 cultivated in greenhouse were collected and stored at -80°C. 100 mg tissues were used for total RNA extraction via RNeasy plant mini kit (Qiagen) based on TRIzol RNA isolation protocol. Quantity and quality of the extracted RNA was determined using a BIOSPEC-NANO spectrophotometer (Shimadzu, Kyoto, Japan) and agarose gel electrophoresis. It was purified using the PolyATract mRNA isolation system IV (Promega, Madison, WI, USA) and then it was used to synthesize the full-length cDNA using ZAP-cDNA synthesis kit (Stratagene, Santa Clara, CA, USA). Finally, cDNA was fragmented by nebulization using an Agilent 2100 BioAnalyzer (Waldbronn, Germany) for library construction with a mean fragment size of about 600 bp.

### Library preparation

For the generation of DNA library to be used for Genome Sequencer FLX titanium (GS-FLX, Roche, Mannheim, GE), approximately, 1 μg of the DNA was used. The cDNA fragments ends were blunted and two short adapters were ligated to both the ends following the standard procedures. The adaptors provided priming sequences for amplification and sequencing of the sample library fragments as well as 'the sequencing keys’, a short sequence of 4 nucleotides. The sequencing key released the unbound strand of each fragment (with 5’ Adaptor A) following repair of any nicks in the double-stranded library. The single stranded template DNA (sstDNA) was quantitated, including a functional quantitation to determine the optimal amount of the library to use as input for emulsion-based clonal amplification.

### 454 pyrosequencing

Manufacturer’s instructions were followed for high-throughput sequencing of the constructed libraries (GS FLX Titanium General Library Preparation Kit/emPCR kit/sequencing kit, Roche Diagnostics, http://www.roche.com) using approximately 1 μg of the adaptor-ligated cDNA population sheared by nebulization. Single effective copies of template species from the DNA library to be sequenced were hybridized to DNA capture beads and the immobilized library was re-suspended in the amplification solution. The mixture was emulsified and subjected to PCR amplification. The DNA carrying beads were recovered from the emulsion and enriched after amplification. As part of the enrichment process, the second strands of the amplification products were melted away, leaving the amplified ssDNA library bound to the beads. The sequencing primer was then annealed to the immobilized amplified DNA templates. After amplification, the DNA-carrying beads were set into the wells of a PicoTiterPlate device (PTP) so that each well contains single DNA beads only. The loaded PTP was eventually inserted into the sequencer for pyrosequencing and sequencing reagents were sequentially flowed over the plate. All the information from the wells was captured and recorded simultaneously and processed in real time.

### Transcriptome assembly and annotation

The transcriptome assembly process is required to account for the co-existence of simultaneously highly related but distinct sequences to represent alternative splice variants of the same as well as different alleles from the same or different loci. The eukaryotic gene expression including the alternative splicing makes this complete process very complicated (Modrek & Lee 2002). GS *de novo* Assembler employs a network or graph-based approach to describe the connectivity between assembled contigs. Individual reads are split by introducing breaks into the assembly and these are further used to define the alternative connections between contigs. Assembled contigs are organized into 'isogroups’ which represent all contigs from a given genetic locus. Within each isogroup, contigs can be connected in different permutations (termed 'isotigs’), each of which can be loosely thought of as a specific splice variant or allele (Sloan et al. 2012; http://454.com/downloads/my454/documentation/gs-flx-plus/454SeqSys_SWManual-v2.6_PartC_May2011.pdf).

The reads thus generated were trimmed of low quality, low complexity [poly(A)], and the adaptor sequences and singleton trimming was analyzed using the SeqClean ver. 1.0 and Lucy program ver. 2.19. *De novo* assembly was performed for the delicate mapping of each sequence. Reference ESTs and mRNA data used for comparison between the sequences of two pepper varieties and that of the public transcript were collected from the NCBI database (http://www.ncbi.nlm.nih.gov/Taxonomy/Browser/wwwtax.cgi?mode=Info&id=4072&lvl=3&lin=f&keep=1&srchmode=1&unlock). All the isotigs and singletons were identified by the analysis of sequence assembly and the assembled transcript sequences were functionally annotated using blastx algorithm and non-redundant protein database at NCBI by blast software with a cut-off e-value of 1.0E-3. By the automated blastx analysis the GI accessions of best hits were retrieved, and the GO accessions were mapped to GO terms according to molecular function, biological process and cellular component ontologies (http://www.geneontology.org/).

### Simple sequence repeat detection

All isotigs and singletons separately from both transcriptome data were used to mine the SSR motifs to obtain the information of molecular markers in both the pepper varieties. All non-redundant trimmed sequences were screened for repeat motifs using the Repeatmasker (http://www.repeatmasker.org/), SSRIT (Simple Sequence Repeat Identification) http://www.gramene.org/db/markers/ssrtool using Perl regular expressions to find perfect SSR within the sequences (Temnykh et al. 2001). Sequences containing at least 6 di nucleotide repeats and 4 tri nucleotide repeats or larger were selected as microsatellite. All motifs having continuous uninterrupted repeats were classified as perfect and those containing more than one class of repeats were classified as compound. Mononucleotide repeats were defined both in terms of base pairs and number of repeats. The resulting output was filtered to exclude duplicate SSRs within the same isogroup because multiple isotigs from the same isogroup can share sequences.

## Results

### sstDNA Library construction

The sstDNA libraries were constructed through total RNA isolation, mRNA purification, cDNA synthesis, cDNA fragmentation and adaptor ligation based on the standard protocols from commercial kits. cDNA was fragmented for the production of transcripts fragments with the standard length necessary for 454 pyrosequencing.

### 454 pyrosequencing and sequence assembly

361,671 and 274,269 raw reads with a total length of 164.49 and 124.60 Mb were generated by the 454 GS FLX Sequencer for Saengryeg 211 and Saengryeg 213, respectively (Table 1). These raw sequencing reads were eventually reduced to 351293 (97.13%) and 266703 (97.24%) high quality sequences for both the varieties after filtering of adaptors, and primers. The raw data were deposited in EBI Sequence Read Archive (SRA) under the accession numbers Study_IDs ERP001874 for Saengryeg 211 and ERP001873 for Saengryeg 213, respectively. For Saengryeg 211; 78.17% of the screened reads (282,705) were incorporated into assembled sequences (isotigs), with remaining 5.01% as singletons (18,147). 60,789 reads (16.80% of reads subjected to assembly) were excluded because of being partially assembled (50,330; 13.91%), from repeat regions (111; 0.03%), outliers (5,075; 1.40%), or too short (5,273; 1.45%). The assembly resulted in 23,821 isotigs out of which 18,363 (77.08%) isotigs were assembled by one contig and the average number of contigs per isotigs was 1.4. The isotig N50 length was 1,028 bp, and the number of isogroups was 20,352 (18,317 [90.00%] of these were assembled by one isotig, whereas 18,295 [89.89%] were assembled by one contig, and the mean number of isotigs per isogroup was 1.2). Similarly, in case of Saengryeg 213; 217,905 (79.45%) of the screened reads were incorporated into assembled sequences (isotigs or contigs), with 15,129 (5.51%) singletons remaining. 60,789 reads (16.80% of reads subjected to assembly) were excluded because they were only partially assembled (33,427; 12.18%), from repeat regions (242; 0.08%), outliers (3,699; 1.34%), or too short (3,831; 1.39%). The assembly resulted in 17,813 isotigs out of which 14,556 (81.71%) isotigs were assembled by one contig and the average number of contigs per isotigs was 1.3. The isotig N50 length was 977 bp, and the number of isogroups was 15,781 (14,537 [92.11%] of these were assembled by one isotig, whereas 14,520 [92.01%] were assembled by one contig, and the mean number of isotigs per isogroup was 1.1).

**Table 1:** **454 Pyrosequencing results for the pepper (** ***Capsicum annuum*** **) varieties, Saengryeg 211 and Saengryeg 213**

454 pyrosequencing terms	Saengryeg 211	Saengryeg 213
Reads		
The Number of raw sequencing reads (n)	361,671	274,269
Bases of raw sequencing reads (bp)	164,494,414	124,608,071
The average of read length (bp)	454,818	454,328
Assembled^a^	282,705	217,905
Partial^b^	50,330	33,427
Singletons^c^	18,147	15,129
Repeat^d^	111	242
Outlier^e^	5,075	3,699
Too short^f^	5,273	3,831
The number of bases, Q20 ≤ (bp)^g^	93,272,495	70,812,087
The number of reads, Q20 ≤ (n)	269,198	205,166
**Isotigs**		
Number of isotigs (n)	23,821	17,813
Average count of contigs in the isotigs (n)	1.401	1.317
Largest count of contigs in the isotigs (n)	12	16
The number of isotigs assembled by one contig (n)	18,363	14,556
The total number of bases in the isotig (bp)	20,787,054	14,965,217
The average isotig size (n)	872.636	840.129
N50 isotig size (bp)^h^	1,028	977
The size of the largest isotig (bp)	17,462	7,300
**Singletons**		
Number of singletons after SeqClean (minimum length, 100)	18,147	15,129
Number of singletons after Lucy (minimum length, 100)	17,054	14,310
Number of valid singletons after Lucy	16,958	14,244

^a^The number of read’s bases used in the assembly computation.

^b^Only part of the read was included in the assembly.

^c^The read did not overlap with any other reads in the input.

^d^The read deemed to be from repeat regions.

^e^The read was identified by the GS *De Novo* Assembler as problematic.

^f^The read was too short to be used in the computation.

^g^The quality of read was higher than or equal to 20.

^h^Half of all bases reside in contigs of this size or longer.

### Functional annotations of the transcriptome sequences

Assembled sequences from pooled reads were validated by sequence based alignments against ESTs submitted at NCBI database. Besides, manual assessment of alignment for all transcripts with their blastx hits against protein NR databases at NCBI was also performed.

**Figure 1:**
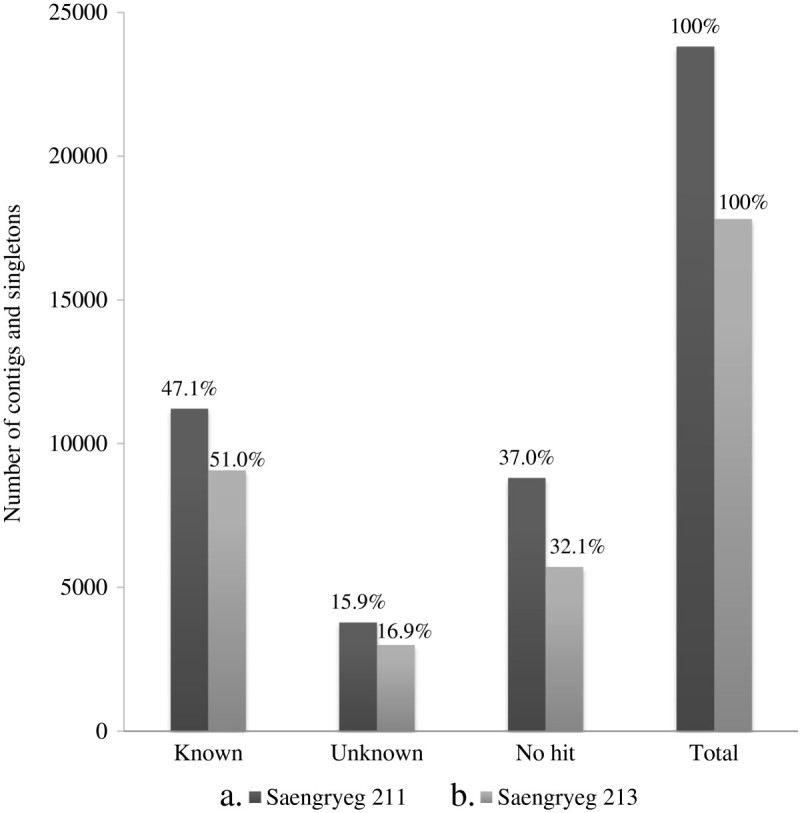
**A comparison of functional annotation result of (a) Saengryeg 211 (b) Saengryeg 213 pepper varieties, using BLAST.**

**Figure 2:**
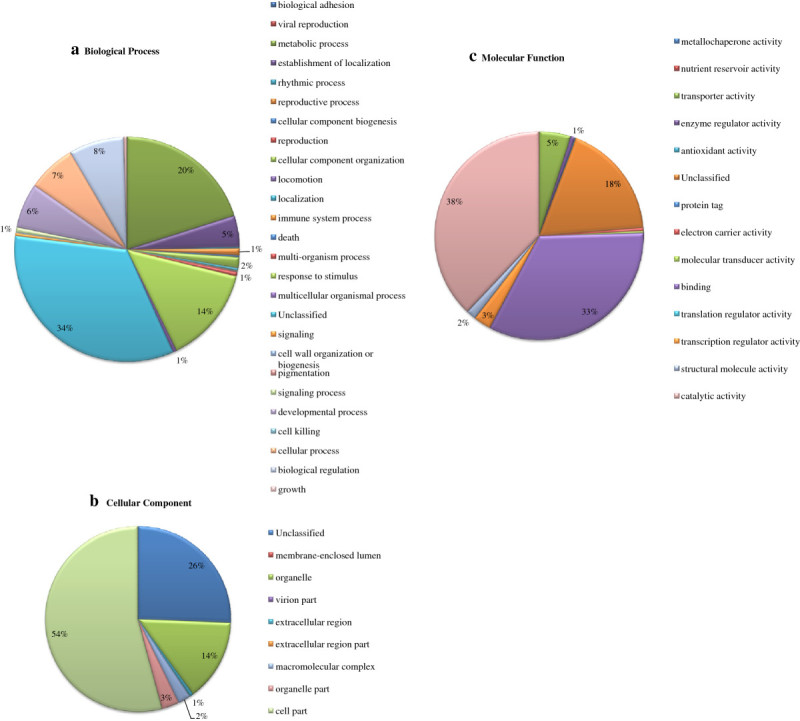
**Classification of variety Saengryeg 211 transcripts into functional categories according to Gene ontology. (a)** Biological Process **(b)** Cellular Component **(c)** Molecular Function.

**Figure 3:**
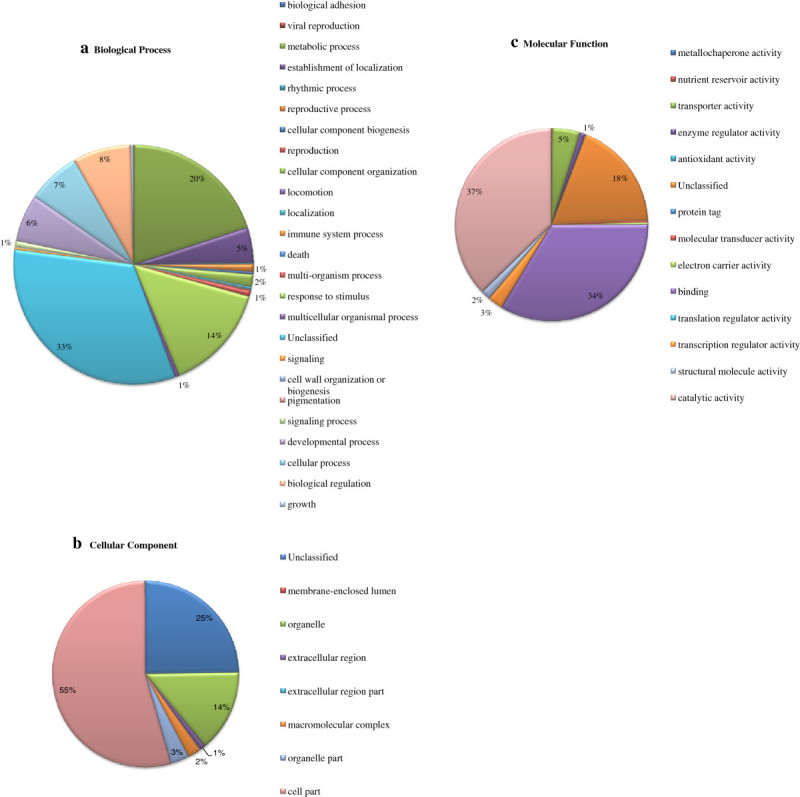
**Classification of variety Saengryeg 213 transcripts into functional categories according to Gene ontology. (a)** Biological Process **(b)** Cellular Component **(c)** Molecular Function.

Out of total 23821 unigenes for Saengryeg 211 and 17813 unigenes for Saengryeg 213, 15007 (62.99%) and 12093 (67.88%) hit at least one significant alignment to existing gene model in blastx searches (Figure 1). The associated hits were searched for their respective GO terms derived from dynamic controlled ontologies to describe the function of genes and gene products. This yielded significant annotations representing the best possible hits which were classified into three major categories namely, biological process, cellular component functions and molecular functions. Of the assigned GO terms, 4555 and 3673 were to known biological processes function, 5032 and 4118 to cellular component function, and 5421 and 4302 to the molecular functions in Saengryeg 211 (Figure 2) and Saengryeg 213 (Figure 3), respectively, indicating a large functional diversity of genes in the transcriptomic data. However, there were still 3,795 and 3,010 sequences unclassified, for both the varieties, respectively. Functional classification of the transcripts in biological process category showed that metabolic process, transport, regulation of biological processes, response to stimulus and cellular process were among the highly represented groups indicating that the plant is undergoing rapid growth and extensive metabolic activity. Genes involved in DNA binding, catalytic and transferase activities were highly represented in molecular function category indicating dominance of gene regulation, signal transduction and enzymatically active processes. Genes involved in other important biological processes such as cell differentiation, communication, transport were also identified through GO annotations. About one third unigenes for both accessions did not hit the highly homologous sequences in the target database, probably representing novel transcripts which could be of great importance for further study.

### SSR Discovery

**Figure 4:**
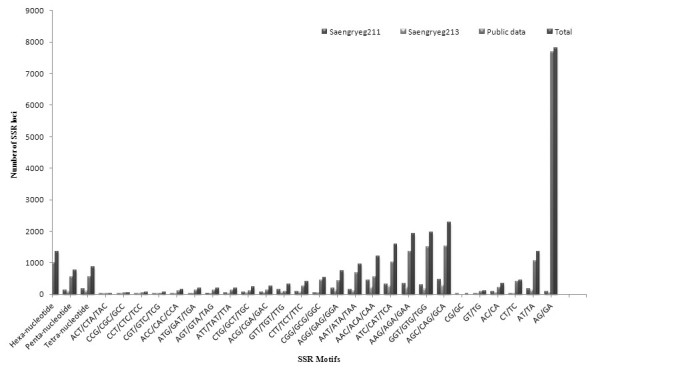
**Frequency distribution of di- and tri-nucleotide repeats in motif sequences of Saengryeg 211 and Saengryeg 213 pepper varieties.**

A total of 3,766 and 2,431 potential SSR motifs, candidates for microsatellite analysis were discovered from the sequenced data for Saengryeg 211 and Saengryeg 213, respectively. Trinucleotide was the most common repeat unit with a frequency of 75.06% and 75.97%, followed by the di (10.43%; 9.62%), hexa (6.02%; 6.17%) and pentanucleotide repeats (3.63%;3.29%) in Saengryeg 211and Saengryeg 213, respectively. The GA repeat (22.3%) was the most frequent repeat motif, followed by CAG (8.7%), TGG (7.6%), GAA (6.5%), and CAT (6.0%) (Figure 4). Mononucleotide SSRs were excluded because of the frequent homopolymer errors found in the Roche 454 pyrosequencing data. The SSRs number of other types with di- and tri-nucleotides motifs was found almost the same, indicating there were some differences existing between Saengryeg 211 and Saengryeg 213.

## Discussion

*De novo* assembly has been performed for the transcriptome analysis, function annotation and development of numerous markers in various plants (Parchman et al. 2010; Wang et al. 2010; Oliver et al. 2011) including pepper to rapidly generate a large amount of sequence data (Lu et al. 2011;2012; Ashrafi et al. 2012; Go’ngora-Castillo et al. 2012; Hill et al. 2013; Liu et al. 2013). Go’ngora-Castillo et al. (2012) and Liu et al. (2013) have introduced the recent study of pepper transcriptome (*Capsicum* transcriptome database).

In the present study, *de novo* transcriptome assembly of these two pepper varieties Saengryeg 211 and Saengryeg 213 were performed by GS Roche Assembler Newbler, which, unlike other assemblers, created isotigs from contigs that are consistently connected by a subset of reads and correspond to alternative transcripts (owing to splicing variants). The reads obtained from the sequencing runs were assembled into isotigs and singletons and were organized into 'isogroups’. Isotigs, which shared the reads, were categorized into the same isogroup. Average lengths of these isotigs were observed to be larger than lengths obtained previously, namely 100 bp for maize (Barbazuk et al. 2007), 247 bp for eucalyptus (Novaes et al. 2008) because of the enhanced performance owing to improvements for longer reads and that of the efficient Roche 454 instrumentation assembler software (Parchman et al. 2010). The majority of pepper EST sequences used in the current project as reference had been first assembled by Kim et al. (2008) in which 22,011 unigenes were assembled with an average consensus sequences length of 1,688 bp. The 454 system generates long sequences but suffers from low sequence depth. However, in recent studies for diploid genome systems in plants, where genome duplication and polyploidy are prevalent, whole genomic samples from large mapping populations are sequenced for complex traits association studies. These have mostly relied on low sequencing coverage at lower depth (2-4x) (Li et al. 2011; Deschamps et al. 2012) which is an attractive strategy for marker discovery from *de novo* characterized samples (Altshuler et al. 2010). The performance of the NGS technologies at low sequence coverage is correlated with per-base sequence coverage uniformity; where Roche 454, with the most uniform coverage, performs the best and suggests that for all the NGS technologies, achieving more uniform sequence coverage would result in considerably higher performance at lower coverage. It provides a powerful and cost-effective alternative to sequencing smaller numbers of individuals at high depth (Flannick et al. 2012).

The assembled sequences were functionally annotated to determine that the majority were involved in proteins with binding function, regulation and metabolism. Two-third of all assembled sequences showed a combination of some known as well as unknown hits. About half of the sequences could be linked with specific functions and the rest were unknown either because no similar sequence was present in the reference database or they matched a protein of unknown function. Approximately one-third of the assembled sequences had no hits and could not be assigned a specific functional annotation. It has been reported that the ability to detect significant sequence similarity partially depends on the length of the query sequence, many of the short sequencing reads obtained using next-generation technology cannot be matched to known genes (Lu et al. 2011). Moreover, the part of sequences showing no hits plausibly included alternative splice variants, novel gene products, and differentially expressed genes, which are of great importance for further research. The transcriptome assembly of two pepper parental lines (CM334 and Taean) and their hybrid line (TF68) has been carried out by Lu et al.^2011^;^2012^ using the GS-454 FLX Titanium to sequence mRNA that was collected from fruits of greenhouse-grown peppers. After the transcriptome assembly, the functional annotation of these contigs determined the involvement of majority of contigs in proteins with binding function, regulation and metabolism. These results are similar to our transcriptome assembly and functional annotation.

A total of 3,766 and 2431 potential SSR markers were identified from these two varieties of *C. annuum* genome sequences, respectively. The tri-nucleotide repeats were found to be the maximum which are more frequently detected in coding regions (Yu et al. 2011). These repeats are generally more robust since they are reported to give fewer “stutter bands” than those based on dinucleotide repeats and these trinucleotide repeats in particular have been demonstrated to be highly polymorphic and stably inherited (Yang et al. 2012). While the tri- and dinucleotides repeats mostly contributed to the major proportion of SSRs in these two varieties, a very small share was contributed by mono-, tetra-, penta- and hexa-nucleotide repeats. A similar trend was observed in other species (Sonah et al. 2011). Based on the variety of SSRs identified in this study, the future SSR marker optimization may be best focused on those comprising trinucleotide repeats. Detection of mutants by comparative marker analysis in these non-pungent and pungent chilli peppers might render new information on structural or regulatory genes that participate in capsaicinoid biosynthesis (Liu et al. 2013). It could lay a platform for better understanding the metabolic pathways in chilli pepper. However, all the predicted molecular markers need to be validated.

## Conclusions

This study provides a significant strategy of a) *de novo* transcriptome assembly which can be potentially used for any species for broad characterization of genes; b) functional annotation of the assembled transcripts to number of putative genes related to numerous metabolic and biochemical pathways and c) identification of large numbers of high quality SSRs with diverse motifs, which upon validation could facilitate the identification of polymorphisms within the species. Combined with some recent previous works on pepper the large number of potential SSRs detected here provides a plethora of potential markers that may prove useful in multiple applications including genetic mapping and QTL analyses. Nevertheless, the ultimate goal remains to use the available genetic resources to develop new pepper varieties with higher yields, better flavours and more resistance to biotic as well as abiotic stresses.

## Authors’ original submitted files for images

Below are the links to the authors’ original submitted files for images. Authors’ original file for figure 1 Authors’ original file for figure 2 Authors’ original file for figure 3 Authors’ original file for figure 4 Authors’ original file for figure 5
