# Supplementary figures and images for: De novo transcriptome assembly and novel microsatellite marker information in Capsicum annuum varieties Saengryeg 211 and Saengryeg 213
Source: Bot Stud. 2013 Nov 21;54:58. doi: 10.1186/1999-3110-54-58 (PMC5430321; doi:10.1186/1999-3110-54-58)

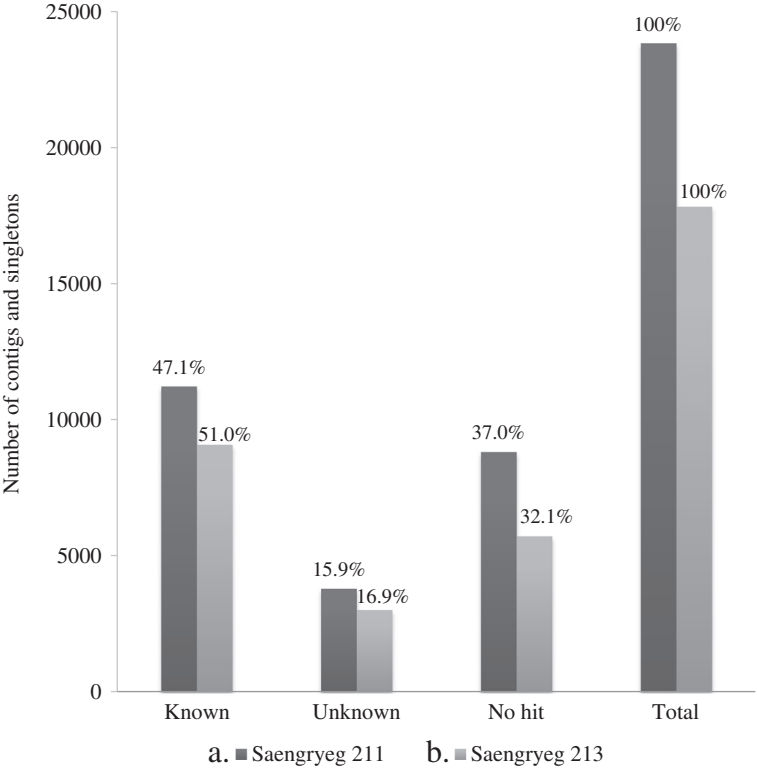

Supplement: Supplementary file 1 — Authors’ original file for figure 1 [file 40529_2013_50_MOESM1_ESM.pdf]

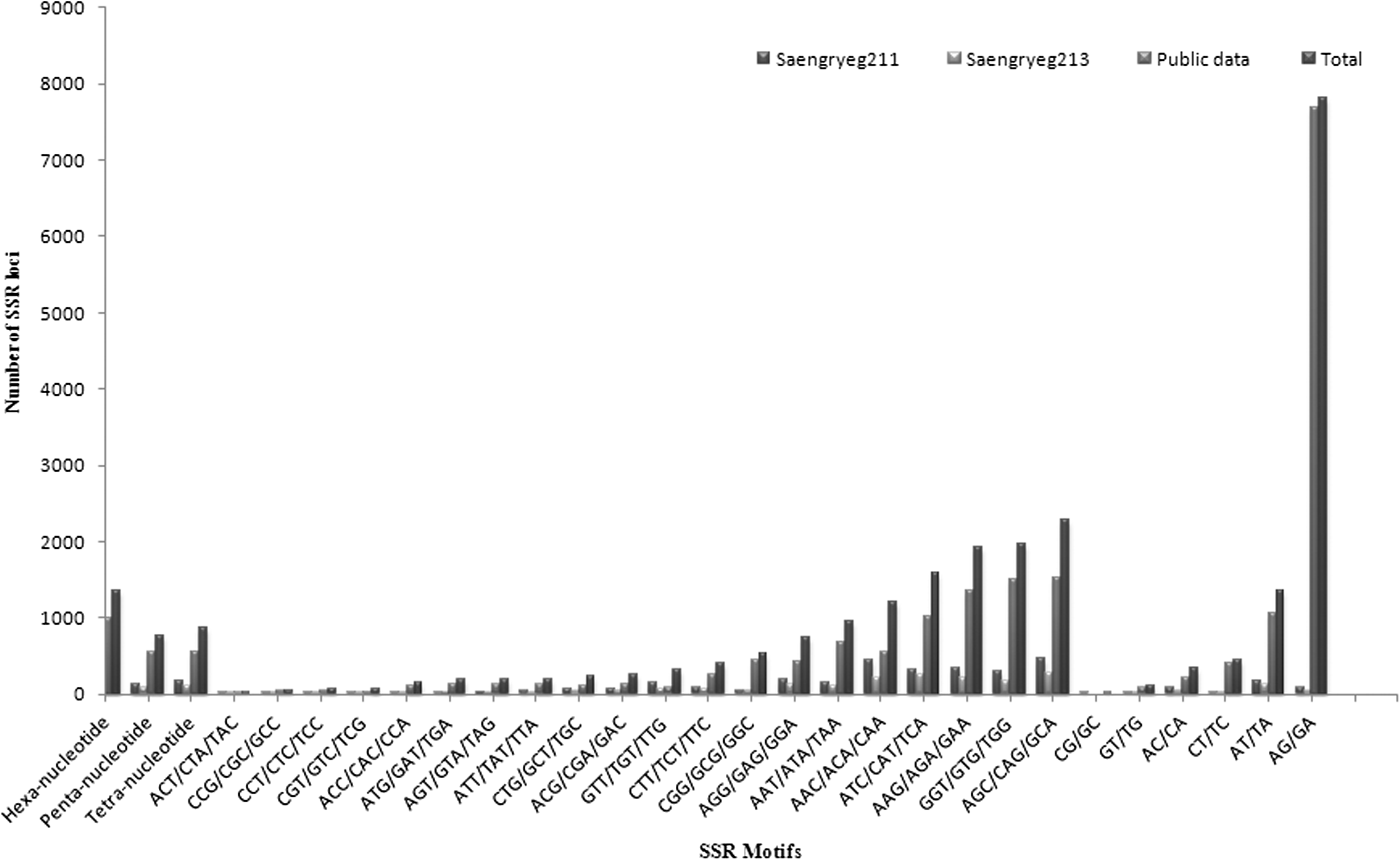

Supplement: Supplementary file 4 — Authors’ original file for figure 4 [file 40529_2013_50_MOESM4_ESM.tif]

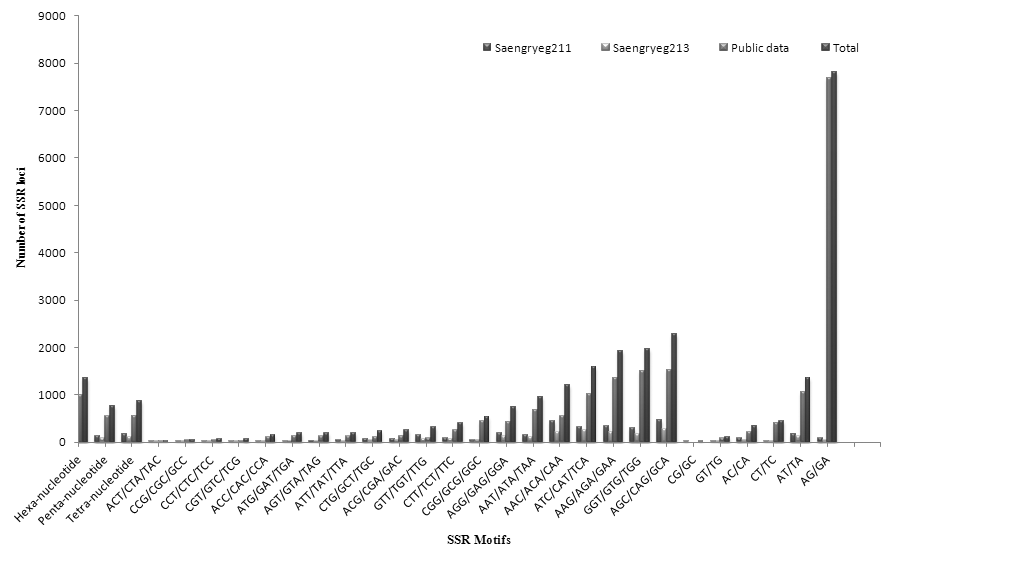

Supplement: Supplementary file 5 — Authors’ original file for figure 5 [file 40529_2013_50_MOESM5_ESM.tiff]
