# Supplementary material for: De novo transcriptome assembly and novel microsatellite marker information in Capsicum annuum varieties Saengryeg 211 and Saengryeg 213
Source: Bot Stud. 2013 Nov 21;54:58. doi: 10.1186/1999-3110-54-58 (PMC5430321; doi:10.1186/1999-3110-54-58)
Supplement: Supplementary file 2 — Authors’ original file for figure 2 [file 40529_2013_50_MOESM2_ESM.pdf]

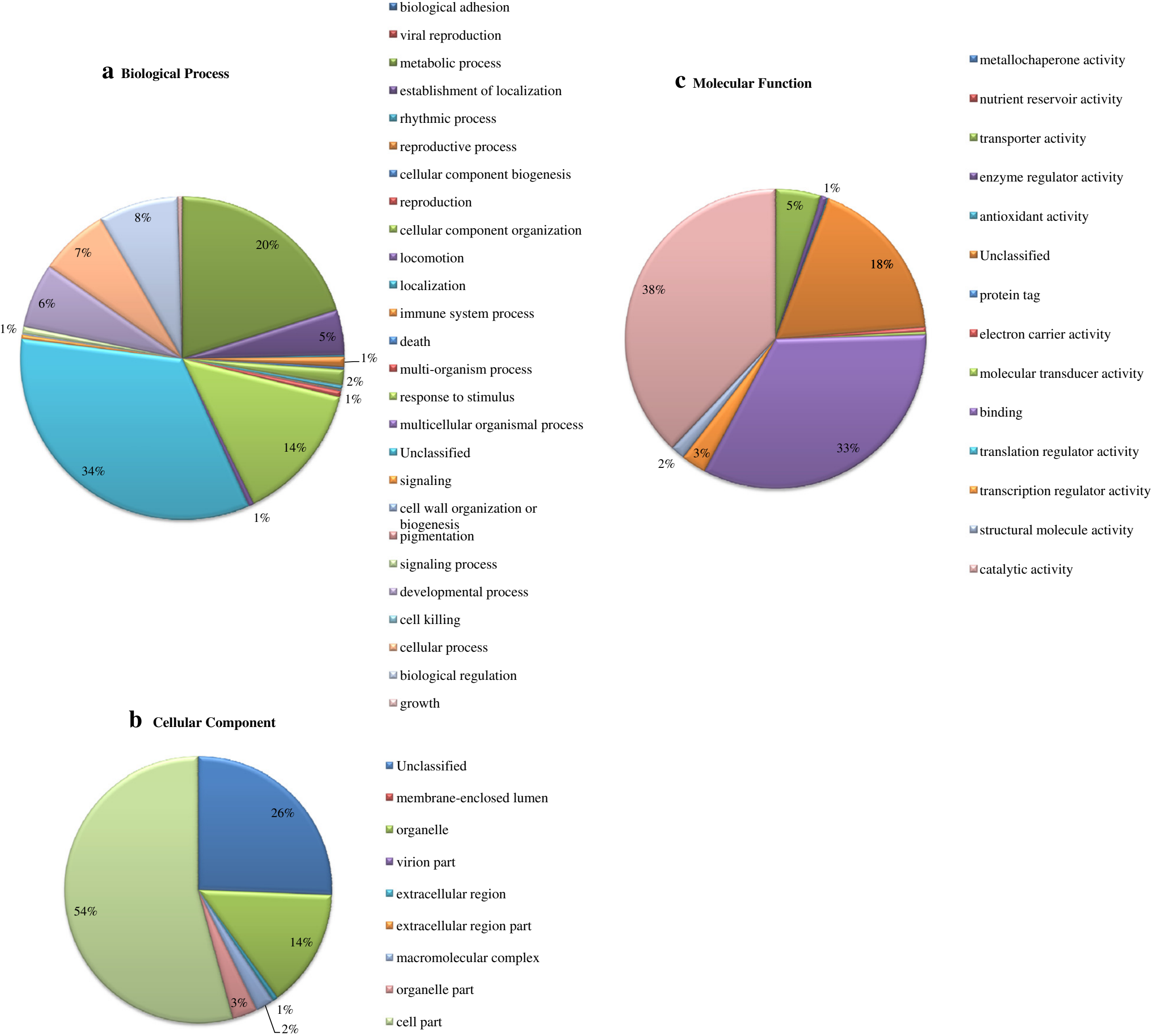
a Biological Process
biological adhesion
viral reproduction
metabolic process
establishment of localization
rhythmic process
reproductive process
cellular component biogenesis
reproduction
cellular component organization
locomotion
localization
immune system process
death
multi-organism process
response to stimulus
multicellular organismal process
Unclassified
signaling
cell wall organization or biogenesis
pigmentation
signaling process
developmental process
cell killing
cellular process
biological regulation
growth
20%
5%
1%
2%
1%
14%
1%
34%
1%
6%
7%
8%
b Cellular Component
Unclassified
membrane-enclosed lumen
organelle
virion part
extracellular region
extracellular region part
macromolecular complex
organelle part
cell part
26%
14%
1%
2%
3%
54%
c Molecular Function
metallochaperone activity
nutrient reservoir activity
transporter activity
enzyme regulator activity
antioxidant activity
Unclassified
protein tag
electron carrier activity
molecular transducer activity
binding
translation regulator activity
transcription regulator activity
structural molecule activity
catalytic activity
5%
1%
18%
33%
3%
2%
38%
